# Smartphone-Based Monitoring of Quality of Life and Adverse Events After Neurosurgery: Prospective Cohort Study

**DOI:** 10.2196/100934

**Published:** 2026-09-09

**Authors:** Alexis Paul Romain Terrapon, Erik Schulz, Athina Firtinidou, Silvio Heinig, Lorenzo Bertulli, Felix Corr, Meltem Gönel, Maria Nikolaeva, Thomas Petutschnigg, Sivani Sivanrupan, Jakob Heimer, Menno R Germans, Christian Zweifel, Andreas Raabe, Philippe Schucht, Luca Regli, Marian Christoph Neidert, Oliver Bozinov, Linda Bättig, Martin Nikolaus Stienen

**Affiliations:** 1 Department of Neurosurgery, Inselspital, Bern University Hospital, University of Bern, Rosenbühlgasse 25, Bern, 3010, Switzerland, 41 316640239; 2 Department of Neurosurgery, Cantonal Hospital of St. Gallen, H-OCH Health Ostschweiz, St.Gallen, Switzerland; 3 Department of Neurosurgery and Clinical Neuroscience Center, University Hospital and University of Zurich, Zürich, Switzerland; 4 Clinical Trials Unit, Cantonal Hospital of St. Gallen & Medical School of St. Gallen, H-OCH Health Ostschweiz, St.Gallen, Switzerland; 5 Department of Surgery, Neurosurgical Unit, Kantonsspital Graubünden, Chur, Switzerland; 6 Faculty of Medicine, University of Basel, Basel, Switzerland

**Keywords:** patient-reported outcome measures, PROMs, smartphone app, mobile, complications, complication, classification of complications, classification of neurosurgical complications, neurosurgery, digital health, surgery, postoperative, recovery

## Abstract

**Background:**

Postoperative outcome assessment is often based on discrete follow-up visits, limiting characterization of individual recovery trajectories, and the timely identification of adverse events (AEs). Longitudinal smartphone-based monitoring may overcome these limitations by enabling frequent, resource-efficient collection of patient-reported outcomes and complications throughout recovery. Such data may provide a more patient-centered understanding of the postoperative course and complement conventional clinical surveillance.

**Objective:**

This study aimed to evaluate the feasibility of smartphone-based longitudinal monitoring of quality of life, subjective well-being, and AEs after elective neurosurgery and compare postoperative recovery trajectories and agreement between patient- and clinician-reported AEs.

**Methods:**

This interim analysis of a prospective cohort study included adult patients undergoing elective lumbar decompression, lumbar fusion, supratentorial craniotomy, or infratentorial craniotomy at a Swiss tertiary referral center between June 2023 and January 2025. Participants used a smartphone app to longitudinally report subjective well-being (Subjective Well-Being Index; 0‐10), quality of life (EQ-5D-5L), and AEs for up to 1 year postoperatively. Complications were self-reported using the Therapy-Disability-Neurology (TDN) classification and retrospectively adjudicated by physicians. Descriptive analyses assessed data density, engagement, and concordance between patient- and clinician-reported events. Mixed-effects models were used to evaluate factors associated with postoperative well-being.

**Results:**

Of the 100 enrolled patients (median age 64.0, IQR 52.95‐71.6 years; n=45, 45% women), 86 (86%) provided postoperative data. During a median follow-up of 3.2 (IQR 0.2‐11.3) months, participants submitted 4354 longitudinal well-being entries. Patients reported 22 unique AEs, whereas physicians identified 44 AEs, with overlap for 9 (20.5%) events. Most physician-reported AEs were mild (30/44, 68.2%; TDN grade 1‐2), and no grade 4 or 5 events occurred. Patient-reported AEs primarily reflected symptomatic and functional impairments, whereas physician-reported events more often included clinically detected or subclinical findings. In mixed-effects models, time since surgery was associated with improved well-being, and no other factors were statistically significant.

**Conclusions:**

Smartphone-based postoperative monitoring was feasible in this elective neurosurgical cohort and generated dense longitudinal patient-reported data beyond routine follow-up. Patient and clinician AE reporting captured partly distinct aspects of postoperative recovery, suggesting that smartphone-based self-reporting may complement rather than replace clinical surveillance.

## Introduction

Understanding and analyzing the impact of surgery and adverse events (AEs) on the subjective well-being of patients is of paramount importance as it provides meaningful input for informed risk-benefit discussions. Current methods in outcome research are often static, resource-intensive, and prone to missing data issues. This results in a poor understanding of the normal postoperative course, which further complicates the consistent identification and reporting of AEs as they are typically defined as deviations from that course [1].

Another major challenge lies in the lack of standardized AE grading systems that reflect patients’ subjective experience. The most widely used perioperative AE grading system is the therapy-based Clavien-Dindo grading scale (CDG), which fails to detect the severity of AEs that are not treated by means of pharmacotherapy and/or surgery [1,2]. This is a key limitation as new neurological deficits are frequent AEs with potentially dramatic consequences on quality of life (QoL), yet they are considered as low grade in therapy-based grading systems such as the CDG. A more recent adaptation, ClassIntra, incorporates disability as a severity criterion but does so too narrowly for neurosurgery and is restricted to intraoperative AEs only [3]. Other classifications developed specifically for the neurological setting suffer from similar limitations [4-9].

To address this, our group proposed the Therapy-Disability-Neurology (TDN) grade [10], which incorporates therapeutic interventions (as in the CDG) but also includes associated neurological deficits and the resulting disability. Previous validation studies have demonstrated robust reliability and a correlation with functional outcome, financial burden, hospital length of stay, and subsequent mortality [10-14]. However, the relationship between AE severity and patient-reported QoL has not yet been established [15], and there remains a need for scalable, longitudinal data collection tools that can capture both clinical events and subjective well-being.

The increasing use of smartphones across all age groups offers unprecedented opportunities for scalable, real-time outcome assessment. Previous studies have explored the use of mobile apps for monitoring recovery and complications after neurosurgery and other surgical specialties, highlighting both their potential and current gaps in validation, user engagement, and reporting quality [16]. We developed a smartphone app (OP Tracker) in collaboration with the webgearing AG agency to assess patients’ well-being in a standardized and longitudinal fashion. The app collects longitudinal self-reported data (Subjective Well-Being Index; SWI) at predefined time points before and after surgery. It also records the type of surgery and the description and severity of AEs (using both the CDG and TDN grade), along with a standardized QoL questionnaire (EQ-5D-5L) [17]. A simplified version tested in a hypothetical scenario with neurosurgeons showed good acceptance and technical feasibility [18].

This digital approach enables high-frequency, resource-efficient outcome assessment and may allow for earlier detection of postoperative deterioration. In this interim report, we present descriptive results from the early phase of an ongoing multicenter, prospective observational study using the OP Tracker app to assess patients’ QoL and perioperative AEs before and up to 1 year after neurosurgery. The primary aim was to evaluate the feasibility of real-time, smartphone-based data collection. Specifically, we examined recruitment dynamics, data completeness, accuracy in the detection and grading of AEs, and early trends in patient-reported outcomes to determine the practicality and potential of this digital approach for broader implementation and future outcome modeling.

## Methods

### Study Design

This report presents an interim analysis of a prospective multicenter observational study designed to evaluate the feasibility and utility of smartphone-based longitudinal monitoring of subjective well-being (SWI, measured using a graphic rating scale with integer values from 0 to 10) and AEs following neurosurgical procedures. The SWI is a single-item rating scale that we developed for low-burden, repeated smartphone-based assessment of overall subjective well-being. Its format is conceptually similar to that of the numeric rating scales from 0 to 10 commonly used for subjective symptoms such as pain. It was chosen to enable frequent longitudinal measurements without the burden of repeatedly completing longer questionnaires. The SWI has not undergone formal validation. The overarching aim of the complete study was to develop a statistical model quantifying the association between patient-reported well-being and relevant clinical variables, thereby characterizing the normal postoperative trajectory and how it may vary depending on clinical factors, including the type and severity of AEs. The present analysis emphasizes feasibility and descriptive statistics. Patient enrollment began in June 2023 at the Cantonal Hospital of St. Gallen (now part of HOCH Health Ostschweiz), a tertiary academic hospital in Switzerland. This interim analysis is restricted to participants enrolled at this site from June 2023 to January 2025. Additional recruitment has since commenced at 3 other major neurosurgical centers in Switzerland: University Hospital Zurich, Cantonal Hospital Graubünden, and Inselspital Bern. These results thus represent the early phase of a larger Swiss multicenter study.

### Eligibility Criteria and Study Procedure

Patients scheduled for one of the following four predefined elective neurosurgical procedures were screened: (1) lumbar decompression with or without sequestrectomy, discectomy, or foraminotomy (including single- or multiple-level procedures); (2) lumbar transpedicular instrumentation and fusion (with or without interbody cages, including thoracolumbar or extension to the pelvis); (3) supratentorial craniotomy; and (4) infratentorial craniotomy. Patients were eligible for inclusion if they were at least 18 years of age, able to consent, willing to provide data up to 1 year after surgery, and in possession of and capable of using a smartphone (Android or iOS); had the necessary language (German) and cognitive skills to use the OP Tracker app; and were able to provide a preoperative SWI and QoL assessment. Exclusion criteria included pregnancy, foreseeable difficulties using the OP Tracker app, presence of a condition that hindered the baseline preoperative assessment, and health conditions that rendered inclusion unsafe (eg, untreated ruptured intracranial aneurysm). Emergency surgical patients were excluded unless surgery was scheduled at least one day after admission, allowing time for proper inclusion and baseline data entry.

Following written informed consent, the OP Tracker smartphone app was installed on the participants’ devices. A study physician entered the date and type of surgery into a secure web interface, which generated a unique participant number (UPN). This UPN was used to pseudonymize the app-based data. Baseline assessments, including demographic information, surgical indication, and comorbidities, were recorded by the study team in a separate spreadsheet. Within the app, the following data were collected: date of surgery, category of surgery, SWI (rated 0-10), EQ-5D-5L questionnaire, and AEs. Each entry was time-stamped. One item of the EQ-5D-5L records self-rated QoL on a visual analog scale ranging from 0 to 100. To ensure consistency with the SWI, these values were linearly transformed to the range of 0 to 10 and incorporated as additional SWI entries. AEs were recorded using predefined categories, with an additional free-text option for events not represented by these categories, and their severity was graded by the patient using the TDN grade (Table 1) [10].

**Table 1.** The Therapy-Disability-Neurology (TDN) grading^a^.

TDN grade	Therapy dimension	Disability dimension	Neurology dimension
1	AE^b^ without the need for treatment other than antiemetics, antipyretics, analgetics, diuretics, electrolytes, physiotherapy, and bedside wound opening	AE not impacting daily life activities	AE not causing any new neurological deficit
2	AE requiring pharmacological treatment, including blood transfusion and total parenteral nutrition	AE hindering at least one activity of daily living	AE causing any new neurological deficit
3	AE requiring an invasive procedure	AE hindering walking or preventing the patient from attending to their own bodily needs	—^c^
4	Life-threatening AE requiring management in intensive care	AE leaving the patient bedridden, in need of constant help, or incontinent	—
5	AE resulting in death	—	—

a The TDN grade is a multidimensional classification of neurosurgical complications [10]. The “therapy” dimension encompasses the Clavien-Dindo classification by Dindo et al [1] and the classification by Landriel Ibañez et al [4]. The “disability” dimension is based on the modified Rankin Scale [19]. The TDN grade is equal to the worse dimension. For example, an AE requiring an invasive procedure (therapy dimension=3, T3) but not impacting daily life activities (D1) or causing any new neurological deficit (N1) is classified as TDN grade 3 (therapy grade 3, disability grade 1, and neurology grade 1=TDN 3; T3D1N1).

b AE: adverse event.

c Not applicable.

**Figure 1.**
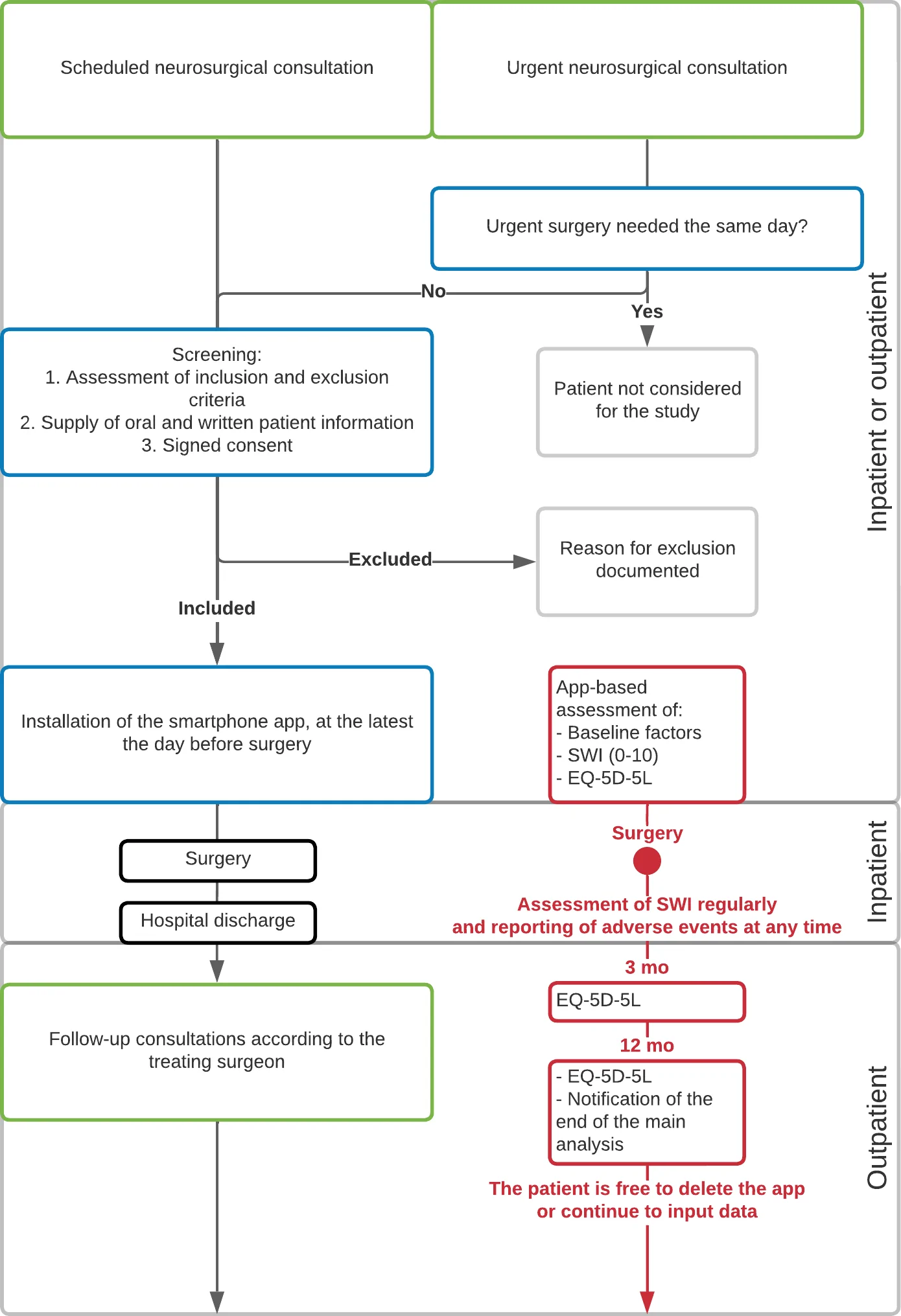
Flowchart of study design and patient pathway from inclusion to follow-up. The flowchart illustrates the study inclusion process and follow-up schedule. Patients were screened no later than the day before surgery. After providing informed consent and installing the app, baseline data including subjective well-being (Subjective Well-Being Scale [SWI]; 0‐10) and quality of life (EQ-5D-5L) were recorded. Postoperative follow-up included regular SWI input and adverse event reporting through the app. The EQ-5D-5L was reassessed at 3 and 12 months. After 12 months, patients may choose to delete the app or continue using it. Standard clinical follow-up was conducted at the treating surgeon’s discretion.

Patients received automated app reminders for data entry: daily in the first 2 weeks postoperatively (starting from the first postoperative day), every 2 days from day 15 to day 42, twice weekly up to 3 months, weekly up to 6 months, and biweekly up to 1 year after surgery. AEs could be reported at any time. If an AE was reported, the reminder frequency was reset to daily for 14 days. EQ-5D-5L reassessments were scheduled at 3 and 12 months. The full study pathway, including enrollment and follow-up schedule, is illustrated in Figure 1.

### Data Collection and Management

A dedicated screening log was maintained daily by the authors. To identify candidates as early as possible, a study nurse screened the neurosurgical operation schedule weekly, entering patients planned for 1 of the 4 predefined procedures into a secure study spreadsheet. This screening log was updated daily by the investigators to account for short-notice scheduling changes, ensuring that eligible patients were not missed. Recruitment took place either in the outpatient setting (at the time of surgical planning) or after hospital admission provided that surgery was not planned for the same day. For each enrolled patient, baseline demographic and clinical information (sex, age, surgical indication, and planned procedure) was recorded in a secure, pseudonymized (UPN) spreadsheet. The first data entry on the OP Tracker app was completed together with the investigators to ensure usability and correct data entry by the patient. Subsequent data entries were completed independently by the patient. Data collected via the app were automatically encrypted and uploaded to a secure Swiss-based server. Investigators accessed the data through a password-protected, web-based platform. AEs were defined as any deviation from the expected perioperative course, including intraoperative and postoperative events. They were later assessed and graded according to the TDN classification by 2 study physicians through retrospective review of medical records. A similar methodology was recently validated, with excellent inter- and intrarater reliabilities [12]. The screened medical records comprised operative reports, discharge summaries, and outpatient follow-up documentation. Laboratory and imaging data were not systematically rescreened using predefined diagnostic thresholds; AEs were recorded when explicitly documented by the treating physicians.

### Outcomes, Sampling, and Bias

The primary objective of this interim study was to assess the feasibility of longitudinal, smartphone-based monitoring in a neurosurgical patient population. Feasibility was assessed descriptively based on recruitment rates, participant adherence to data entry, completeness of SWI and EQ-5D-5L data, and reliability of AE reporting via the app. As this was an exploratory feasibility assessment, no predefined quantitative thresholds for successful feasibility were specified. As part of the exploratory analysis, we assessed the suitability of future modeling approaches (mixed-effects models) to capture within- and between-patient variation in SWI values over time. Additionally, patient-reported and physician-adjudicated AEs were compared based on frequency, type, and severity. This feasibility phase did not involve formal sample size calculation. Selection bias was expected as only patients with compatible smartphones and sufficient digital literacy could participate. Recall bias was reduced through frequent notifications and display of prior SWI entries, which may introduce anchoring bias. Additionally, attrition bias is likely to occur as patients experiencing severe complications are more likely to discontinue app use, leading to underreporting of unfavorable outcomes. No external control group was used in this interim study.

### Statistical Analysis

All analyses were performed using R (version 4.4.0; R Foundation for Statistical Computing) [20]. Data were manipulated using *tidyverse* [21] and visualized using *ggplot2* [22], and summary tables were formatted using *gt* [23]. Categorical variables are summarized as frequencies and/or percentages, and continuous variables are summarized using means or medians as appropriate. Analyses were based on available cases; missing data were not imputed. For group comparisons, the Kruskal-Wallis rank-sum test was applied to continuous variables, and the Fisher exact test was used for categorical variables. *P* values are reported for descriptive purposes only, and no adjustment for multiple testing was performed. A *P* value below .05 was considered statistically significant. Using *nlme* [24], a mixed-effects model was constructed to account for differences between patients and the correlation of repeated measurements over time. The influence of time since surgery, age, sex, type of surgery, and both patient-reported and physician-reported AEs on well-being scores was examined. The model included random intercepts and random slopes for time (months) for each patient and accounted for the fact that repeated measurements from the same patient tend to be correlated using an AR1 structure.

### Ethical Considerations

This study was approved by the relevant regional ethics committee (Business Administration System for Ethics Committees [BASEC] 2022-01510; Ethikkommission Ostschweiz [EKOS] 22/131); registered at ClinicalTrials.gov (NCT06352710); and conducted in accordance with the Declaration of Helsinki, the principles of good clinical practice, and the Swiss Human Research Act and Human Research Ordinance. No study-specific clinical interventions were performed, and standard care pathways remained unchanged. All participants gave written informed consent. Additionally, this study complied with the mERA (mobile health evidence reporting and assessment) checklist (Checklist 1) to ensure thorough reporting of digital health research and with the STROBE (Strengthening the Reporting of Observational Studies in Epidemiology) statement for observational studies (Checklist 2) [25,26].

## Results

### Patient Inclusion and Baseline Characteristics

Between June 12, 2023, and January 31, 2025, a total of 626 patients scheduled for neurosurgical procedures at the Cantonal Hospital of St. Gallen were screened for eligibility. Of these 626 patients, 100 (16%) met all inclusion criteria and were included. Reasons for exclusion were preoperative inclusion not feasible due to logistical constraints (eg, same-day surgery; 298/626, 47.6%), anticipated difficulties using the app (85/626, 13.6%), lack of access to a compatible smartphone (60/626, 9.6%), patient refusal to participate (49/626, 7.8%), age below 18 years (7/626, 1.1%), incapacity to provide informed consent (6/626, 1%), current pregnancy (4/626, 0.6%), or unspecified reasons (17/626, 2.7%).

**Figure 2.**
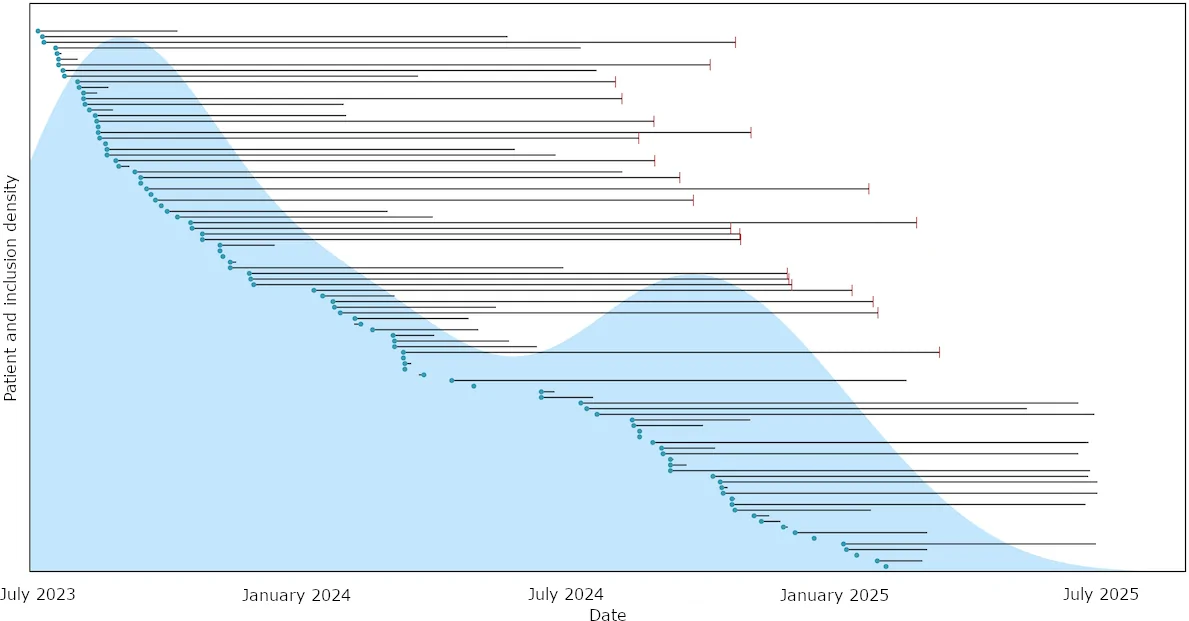
Patient inclusion density and data collection timeline. The blue shaded area represents the smoothed density of included patients, with dots representing the date of individual patient inclusion and the lines representing the length of follow-up. Patients with a full 1-year follow-up are indicated with a vertical red bar at the end of the follow-up period.

The inclusion rate peaked in the third month after study initiation, followed by a decline and then a relatively stable rate over the subsequent months (Figure 2). The drop coincided with an increasing number of patients admitted on the day of surgery, making inclusion impossible due to the requirement for app installation and baseline data entry prior to surgery.

Of the 100 included patients, 68 (68%) underwent spinal surgery, and 32 (32%) underwent cranial surgery. The most common procedures were lumbar fusion (35/100, 35%), followed by lumbar decompression (33/100, 33%). There were 32 craniotomies, of which 28 (87.5%) were supratentorial. The median age at surgery was 64.0 (IQR 52.95-71.6) years, and 45% (45/100) of the participants were female. No significant age difference was observed between subgroups. More female participants underwent spinal fusion, whereas more male participants underwent lumbar decompression or craniotomy. Patients undergoing craniotomy had a markedly higher preoperative QoL compared with those undergoing spinal surgery. These results are summarized in Table 2.

**Table 2.** Baseline characteristics of the study population (N=100)^a^.

	Overall	Lumbar decompression (n=33)	Lumbar fusion (n=35)	Supratentorial craniotomy (n=28)	Infratentorial craniotomy (n=4)	*P* value
Age (y)						.15
Mean (SD)	62.1 (13.9)	62.9 (13.7)	65.6 (12.2)	57.3 (15.0)	58.7 (16.0)	
Median (IQR)	64.0 (52.95-71.6)	62.2 (53.2-71.85)	67.4 (62.5-72.9)	62.1 (49.0-68.7)	61.0 (48.1-69.25)	
Sex, n (%)						.02
Female	45 (45)	11 (33.3)	23 (65.7)	10 (35.7)	1 (25)	
Male	55 (55)	22 (66.7)	12 (34.3)	18 (64.3)	3 (75)	
Preoperative SWI^b^						.001
Mean (SD)	5.6 (2.65)	4.9 (2.5)	4.8 (2.5)	6.9 (2.5)	7.75 (1.0)	
Median (IQR)	5.0 (3.5-8.0)	4.0 (3.0-6.0)	5.0 (3.0-6.0)	7.5 (5.5-9.0)	7.5 (8.0-8.5)	
Preoperative EQ-5D-5L (range −0.661 to 1.000)						<.001
Mean (SD)	0.6 (0.4)	0.5 (0.4)	0.45 (0.3)	0.9 (0.1)	0.9 (0.1)	
Median (IQR)	0.7 (0.4-0.9)	0.6 (0.3-0.8)	0.5 (0.2-0.8)	0.9 (0.9-1.0)	1.0 (0.8-1.0)	

a This table summarizes demographic and baseline characteristics of the 100 patients included in this interim analysis. Patients were grouped according to surgical category. *P* values were computed using the Kruskal-Wallis rank-sum test and Fisher exact test, as appropriate.

b SWI: Subjective Well-Being Index; ranging from 0 (very bad) to 10 (very good).

### Data Completeness

Postoperative data entry through the OP Tracker app was achieved by 86% (86/100) of the participants. The median follow-up period reached 3.2 (IQR 0.2-11.3) months, during which patients submitted a median of 38 (IQR 4-65) SWI entries. The completeness of SWI data was high in the early postoperative period, with 84% (84/100) of scheduled entries completed within the first month following surgery. This declined moderately over time, with completion rates of 59% (59/100) at 3 months, 51.5% (50/97) at 6 months, and 43.7% (31/71) at 12 months. A total of 42% (42/100) of the participants provided more than 50 SWI entries, whereas 35% (35/100) provided less than 10 (Table 3). EQ-5D-5L QoL data were collected at 3 time points. Preoperative responses were obtained from 85% (85/100) of participants, responses at 3 months were obtained from 43% (43/100) of patients, and responses at 12 months were obtained from 32.4% (23/71) of patients. Individual postoperative SWI trajectories for all patients with at least 5 entries are shown in Multimedia Appendix 1.

**Table 3.** Distribution of data entries per patient (N=100)^a^.

Observations, n	Patients, n (%)
1	4 (4)
2	11 (11)
3-4	11 (11)
5-9	9 (9)
10-49	23 (23)
≥50	42 (42)

a This table shows the number of patients stratified by the total number of Subjective Well-Being Index and EQ-5D-5L entries recorded via the OP Tracker app.

### AE Reporting

A total of 30 AEs were self-reported via the OP Tracker app by 15% (15/100) of the participants. Because patients could enter free-text descriptions, similar complications were sometimes described differently, and in some cases, the same AE was reported multiple times. To avoid overestimation, we grouped similar descriptions and counted repeated reports only once per patient, resulting in 22 unique AEs from 15 categories (Table 4).

**Table 4.** Comparison of adverse events (AEs) reported by investigators and patients (N=100)^a^.

AE description	Frequency, n (%)
Investigator reported	
Anemia	6 (6)
Pseudarthrosis and/or implant-related AE	6 (6)
New sensory or motor deficit	4 (4)
Intraoperative dural tear	4 (4)
Wound healing disorder, including infection	2 (2)
Pulmonary embolism	2 (2)
Hypervolemia and/or pleural effusions	2 (2)
Respiratory tract infection	2 (2)
Urinary tract infection	2 (2)
Persistent or aggravated pain	2 (2)
Recurrent disc herniation	1 (1)
SIADH^b^	1 (1)
Acute renal injury	1 (1)
Intraoperative vascular injury	1 (1)
Epileptic seizure	1 (1)
Incisional hernia	1 (1)
Hygroma	1 (1)
Positioning-related hematoma	1 (1)
Sinus vein thrombosis	1 (1)
Nerve root compression due to allogeneic bone material	1 (1)
Dysarthria	1 (1)
Paralytic ileus	1 (1)
Patient reported	
New sensory or motor deficit	4 (4)
Intraoperative dural tear	3 (3)
Urinary tract infection	2 (2)
Persistent or aggravated pain^c^	2 (2)
Wound healing disorder, including infection	1 (1)
Pulmonary embolism	1 (1)
Phlebitis^c^	1 (1)
Respiratory tract infection	1 (1)
Vomiting and diarrhea^c^	1 (1)
Blurred vision^c^	1 (1)
Recurrent disc herniation	1 (1)
SIADH^c^	1 (1)
Postoperative bleeding	1 (1)
Painful hematoma from arterial line^c^	1 (1)
Facial edema^c^	1 (1)

a This table summarizes AEs as prospectively reported by patients via the OP Tracker app and as identified by investigators through retrospective chart review. When a patient reported the same AE multiple times, it was counted only once. Free-text reports that described the same complication in different words were grouped under a common category (eg, all pain-related descriptions were summarized as “Persistent or aggravated pain”).

b SIADH: syndrome of inappropriate antidiuretic hormone secretion.

c Free-text entries that did not correspond to predefined AE categories in the OP Tracker app.

**Figure 3.**
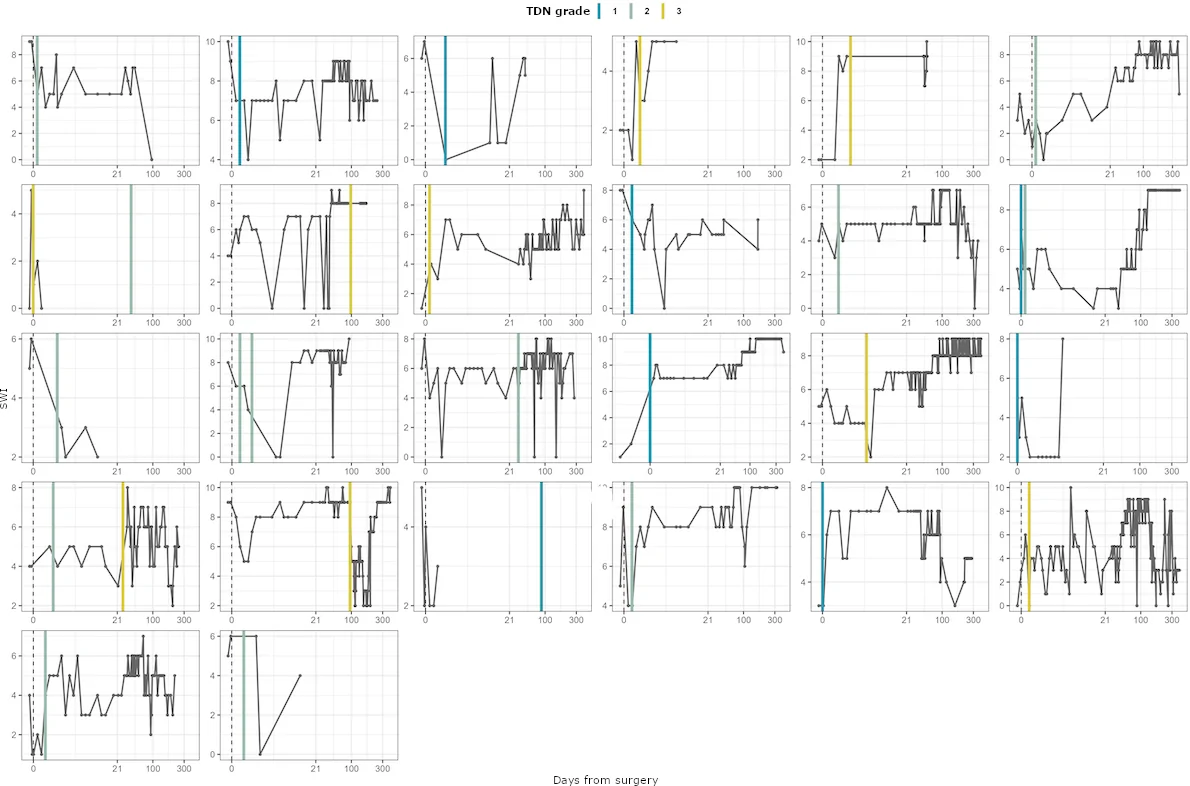
Longitudinal well-being patterns in patients with adverse events (AEs). Each subplot illustrates the Subjective Well-Being Index (SWI; 0=worst; 10=best) over time for all patients with at least 5 SWI entries and ≥1 physician-reported AE. Physician-reported AEs are displayed as vertical bars, with colors indicating AE severity. If the exact date of an AE was unknown, the date of documentation (eg, follow-up consultation) was used. Accordingly, the temporal relationship between AEs and SWI curves should be interpreted with caution, particularly in the late postoperative period. The x-axis represents days from operation (day 0). A piecewise axis transformation was applied to provide high resolution during the early postoperative period while displaying the full long-term follow-up. The scale is linear from day 0 to day 21 and follows a square root function thereafter. TDN: Therapy-Disability-Neurology.

For comparison, hospital records of all included patients were reviewed by 2 investigators. In total, 44 AEs were identified in 36% (36/100) of the patients, and 9 (20.5%) of these events were also correctly reported by patients through the app. In these cases, patients assigned the correct TDN grade in 66.7% (6/9) of cases. The median TDN grade was 2.0 for both physician- and patient-reported AEs (IQR 1.75-3.0 and 2.0-3.0, respectively), with no differences across surgical subgroups. Most investigator-reported AEs were mild (TDN 1 or 2; 30/44, 68.2%), and there were no grade 4 or 5 events. Patients and investigators tended to report different types of AEs: the most frequent patient-reported events were sensory or motor deficits (n=4), intraoperative dural tears (n=3), urinary tract infections (n=2), and persistent or aggravated pain (n=2), whereas the most frequent investigator-reported events were anemia (n=6), pseudarthrosis or implant-related complications (n=6, consisting of 1 cage displacement, 1 nerve root compression by bone graft material, 2 pseudarthrosis, and 2 screw loosenings), sensory or motor deficits (n=4), and intraoperative dural tears (n=4). The longitudinal relationships between patient well-being and AEs are illustrated in Figure 3.

When stratified by surgery type (Table 5), patients who underwent lumbar fusion had the highest rate of investigator-reported AEs (29/35, 82.9%), whereas those who underwent lumbar decompression had the lowest (6/33, 18.2%). Interestingly, lumbar fusion was also the group with the lowest proportion of patient-reported AEs (4/35, 11.4%).

**Table 5.** Adverse event (AE) frequency and severity stratified by type of surgery^a^.

Type of surgery	Investigator reported		Patient reported	
	AEs, n/N (%)	TDN^b^ (1-5), median (IQR)	AEs, n/N (%)	TDN (1-5), median (IQR)
Lumbar decompression	6/33 (18.2)	1.0 (1.0-1.75)	8/33 (24.2)	2.0 (1.0-3.0)
Lumbar fusion	29/35 (82.9)	2.0 (2.0-3.0)	4/35 (11.4)	2.5 (2.0-3.25)
Supratentorial craniotomy	7/28 (25)	2.0 (1.5-2.0)	8/28 (28.6)	2.0 (2.0-2.0)
Infratentorial craniotomy	2/4 (50)	2.0 (2.0-2.0)	1/4 (25)	3.0 (3.0-3.0)

a The table summarizes AEs reported prospectively by patients via the OP Tracker app and those identified retrospectively by investigators through chart review. Median Therapy-Disability-Neurology scores with IQRs are shown to describe AE severity. Repeated patient reports of the same AE were counted only once. If patients assigned different Therapy-Disability-Neurology grades to the same AE, the most severe grade was used.

b TDN: Therapy-Disability-Neurology.

### Mixed-Effects Models

**Figure 4.**
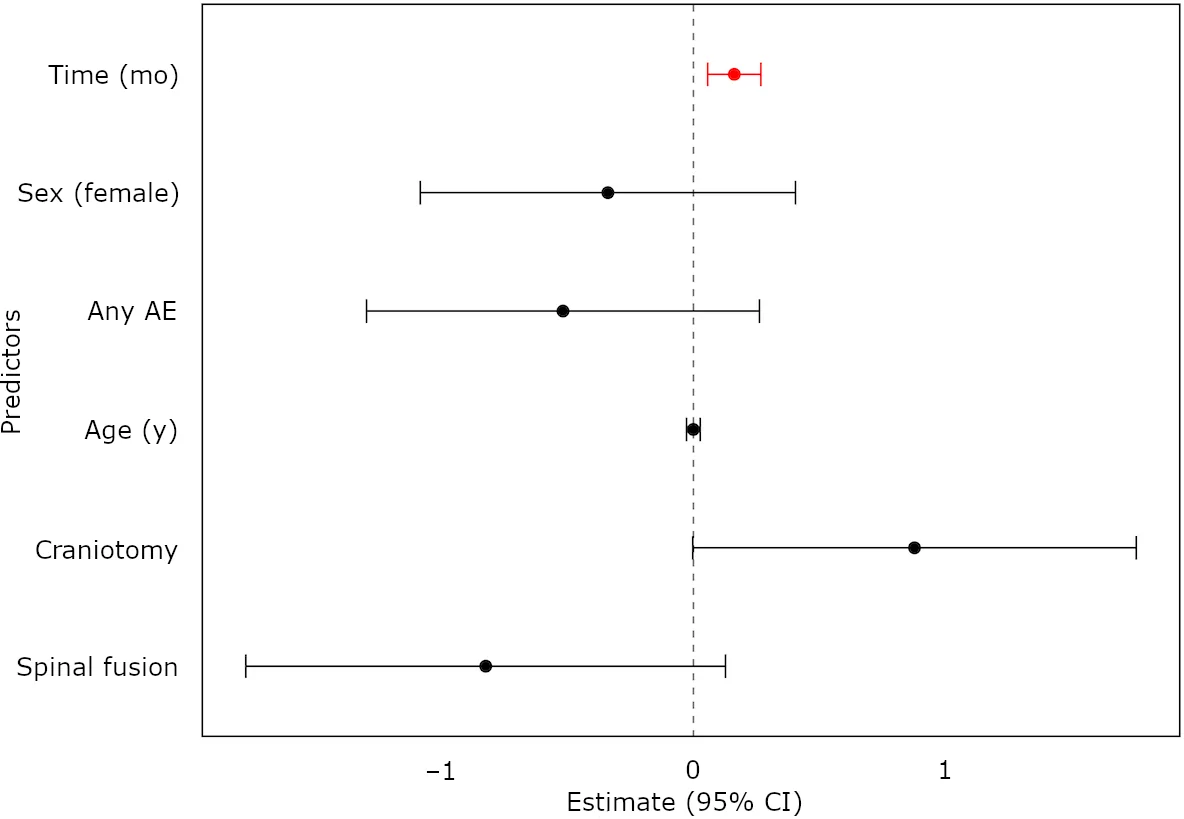
Mixed-effects model of factors influencing postoperative well-being. This figure shows effect estimates from the mixed-effects model evaluating predictors of the Subjective Well-Being Index (SWI). Time since surgery was positively associated with the SWI. No significant associations were observed for other predictors. Significant *P* values are highlighted in red. Error bars represent 95% CIs. AE: adverse event.

A mixed-effects model was computed to evaluate factors influencing postoperative well-being (Figure 4). As expected, time since surgery was positively associated with subjective well-being, reflecting gradual recovery during follow-up. No other factors reached statistical significance as their 95% CIs crossed 0. However, there was a tendency toward lower well-being among female patients, those who underwent spinal fusion, and those who experienced AEs, whereas patients who underwent craniotomy tended to report higher well-being. Importantly, age had no measurable impact on postoperative well-being, suggesting that perceived recovery was not age dependent in this population.

## Discussion

### Key Results

This interim analysis demonstrates the feasibility of smartphone-based longitudinal monitoring of postoperative well-being and AEs in an elective neurosurgical cohort while also identifying challenges related to recruitment and sustained long-term participation. The 100 enrolled patients submitted 4354 QoL entries (mean of almost 50 per patient). Data completeness was highest during the early postoperative period and decreased over time. Patients reported 22 unique AEs compared with 44 identified by investigators, with limited overlap. Most AEs were mild (TDN grade 1-2; 30/44, 68.2%), and none were life-threatening or highly disabling. A preliminary mixed-effects model identified time as the only positive predictor of SWI scores. Together, these findings support the feasibility of patient-driven digital monitoring, demonstrate its potential to provide richer insights than conventional follow-up by complementing physician documentation with the patient perspective, and underscore the importance of sustaining engagement during longer-term follow-up.

### Interpretation and Comparisons to Existing Literature

Postoperative outcome research and complication monitoring continue to face major methodological challenges. First, traditional methods of tracking postoperative outcomes are static and resource intensive (eg, questionnaires during rare follow-up consultations at fixed time points after surgery), leading to sparse data, vulnerability to missing information, and an incomplete understanding of the normal postoperative course. Second, AE reporting and monitoring are hindered by the lack of detection tools as they are usually defined as deviations from the normal postoperative course, which, as stated above, remains unknown. Third, the severity of AEs is usually reported regardless of the disability experienced by patients. The introduction of the TDN classification addressed this gap [10], but the influence of AEs on QoL has not yet been prospectively evaluated. Together, these limitations greatly hinder meaningful outcome assessment, informed patient counseling, and data-driven improvement of medical care.

Addressing these challenges requires new data collection methods that capture patients’ well-being in their own environment and detect deterioration early. In this context, the near ubiquity of smartphones offers a practical platform for such continuous monitoring. Digital health tracking devices, including wearables and smartphone-based sensors, have been shown to detect complications earlier and improve postoperative care in general surgery [16,27] and are increasingly applied in neurosurgical patients as well [28]. Still, no large-scale, patient-driven mobile postoperative monitoring solution has been implemented to our knowledge.

Our solution is an easy-to-use smartphone app that enables close monitoring of life quality fully integrated into patients’ environment. The early findings from our single-center experience offer several insights that may help shape future integration of digital health solutions in the clinical setting. The app enabled us to gather thousands of life quality data points, which would be completely impossible in the usual clinical routine. The successful enrollment and retention of patients validate the practicality of integrating a smartphone app into the care pathway, and the median age of participants (64.0, IQR 52.95-71.6 years) compared adequately with that of a large neurosurgical series from Switzerland [29]. Our results show that many neurosurgical patients are both able and willing to engage in digital health tracking, highlighting the added value of combining patient-reported and clinical data. Engagement was more sustained for the frequently prompted SWI than for the EQ-5D-5L, which was administered only at predefined follow-up time points. Missed EQ-5D-5L prompts were therefore less likely to be recovered through subsequent notifications, suggesting that repeated reminders for infrequently administered outcome measures may improve long-term completeness. Patients and physicians did not always report the same AEs: clinicians often noted laboratory or radiographic findings (eg, anemia, acute kidney injury, and pseudarthrosis) that patients did not perceive as complications, whereas patients mainly reported symptomatic or functionally relevant issues such as neurological deficits, pain, or bed rest after a dural tear. This divergence illustrates that patient-reported outcomes focus on the experienced and functional burden of surgery, complementing physician-based reporting. Using the TDN grade helped bridge these perspectives by prompting patients to consider new deficits and disabilities rather than medical interventions alone. Both patients and physicians predominantly reported low-grade (TDN 1-2) events, reflecting the predominance of minor complications in this elective cohort, and the 20.5% (9/44) overlap indicates that relying on either source alone would miss a substantial part of the postoperative picture. Together, these findings support a hybrid model of outcome monitoring that integrates patient-driven digital reporting with traditional clinical follow-up to achieve a more comprehensive understanding of recovery.

This single-center analysis represents the initial phase of an ongoing multicenter Swiss study aimed at characterizing the postoperative course by analyzing the relationship between patient-reported QoL and clinical variables such as type of surgery and severity of AEs. Subsequent iterations of the OP Tracker app could incorporate configurable procedure- and diagnosis-specific outcome measures. Building on this foundation, future integration of patient-driven self-reporting with passive data streams from wearables, biosensors, and electronic health records could enable real-time, adaptive feedback loops between patients and care teams. Such connected, multimodal approaches have the potential to redefine postoperative care by enabling continuous, personalized recovery monitoring and early detection of complications at scale.

### Limitations

This analysis has several limitations inherent to its design and scope. It included the first 100 patients from a single center, with a predominance of spinal procedures, which may limit generalizability. Because participation required the use of a smartphone, selection bias is likely. Patients with limited digital access or literacy, significant neurological impairment, or poor postoperative recovery may therefore be underrepresented. This is particularly relevant because these patients may also be at increased risk of postoperative complications, limiting the generalizability of our findings to the broader neurosurgical population. In addition, severe or higher-grade AEs are expected to be underrepresented in patient-reported data, particularly when the event itself limits the patient’s ability to interact with the app. This highlights that smartphone-based self-reporting should complement rather than replace clinical surveillance. Similarly, AEs that were asymptomatic or perceived as expected may have been underreported by patients. This is reflected in the lower number of patient-reported AEs compared with physician documentation. However, the parallel physician assessment of AEs mitigates this limitation by providing a more complete overview and showing a similar TDN grade distribution, suggesting that patients can use the system meaningfully for less severe complications. The inclusion of heterogeneous spinal and cranial procedures was appropriate for assessing the feasibility of a broadly applicable monitoring platform, but differences in baseline characteristics, recovery trajectories, and AE profiles limit the interpretation of pooled analyses and the exploratory mixed-effects model. Procedure-specific analyses will require larger subgroup sample sizes. Finally, as an interim single-center analysis, these results should be interpreted as preliminary but supportive of the feasibility and potential of patient-driven digital monitoring.

### Conclusions

In this interim analysis, smartphone-based postoperative monitoring was feasible and engaging for neurosurgical patients, generating high-density, patient-centered data that complement traditional clinical follow-up. The integration of patient-reported well-being and AE tracking offers a richer, more nuanced view of recovery and highlights the potential of digital tools to close long-standing gaps in outcome research. As the multicenter study progresses, this approach may pave the way toward data-driven, patient-centered systems for continuous and personalized monitoring of postoperative recovery, although its impact on clinical decision-making and patient outcomes remains to be established.

## Supplementary material

10.2196/100934 Multimedia Appendix 1 Longitudinal well-being patterns. Each subplot illustrates the Subjective Well-Being Index (SWI; 0=worst; 10=best) over time for all patients with at least 5 SWI entries. The x-axis represents days from operation (day 0). A piecewise axis transformation was applied to provide high resolution during the early postoperative period while displaying the full long-term follow-up. The scale is linear from day 0 to day 21 and follows a square root function thereafter.

10.2196/100934 Checklist 1 mERA checklist.

10.2196/100934 Checklist 2 STROBE checklist.

## Abbreviations

AE: adverse event
BASEC: Business Administration System for Ethics Committees
CDG: Clavien-Dindo grading scale
EKOS: Ethikkommission Ostschweiz
mERA: mobile health evidence reporting and assessment
QoL: quality of life
STROBE: Strengthening the Reporting of Observational Studies in Epidemiology
SWI: Subjective Well-Being Index
TDN: Therapy-Disability-Neurology
UPN: unique participant number
