# Supplementary material for: Smartphone-Based Monitoring of Quality of Life and Adverse Events After Neurosurgery: Prospective Cohort Study
Source: J Med Internet Res. 2026 Sep 9;28:e100934. doi: 10.2196/100934 (PMC13557324; doi:10.2196/100934)

Multimedia Appendix 1 – Longitudinal well-being patterns

Each subplot illustrates the Subjective Well-being Index (SWI; 0 = worst, 10 = best) over time for all patients with at least five SWI entries. The x-axis represents days from operation (Day 0). A piecewise axis transformation was applied to provide high resolution during the early post-operative period while displaying the full long-term follow-up. The scale is linear from Day 0 to Day 21 and follows a square root function thereafter.

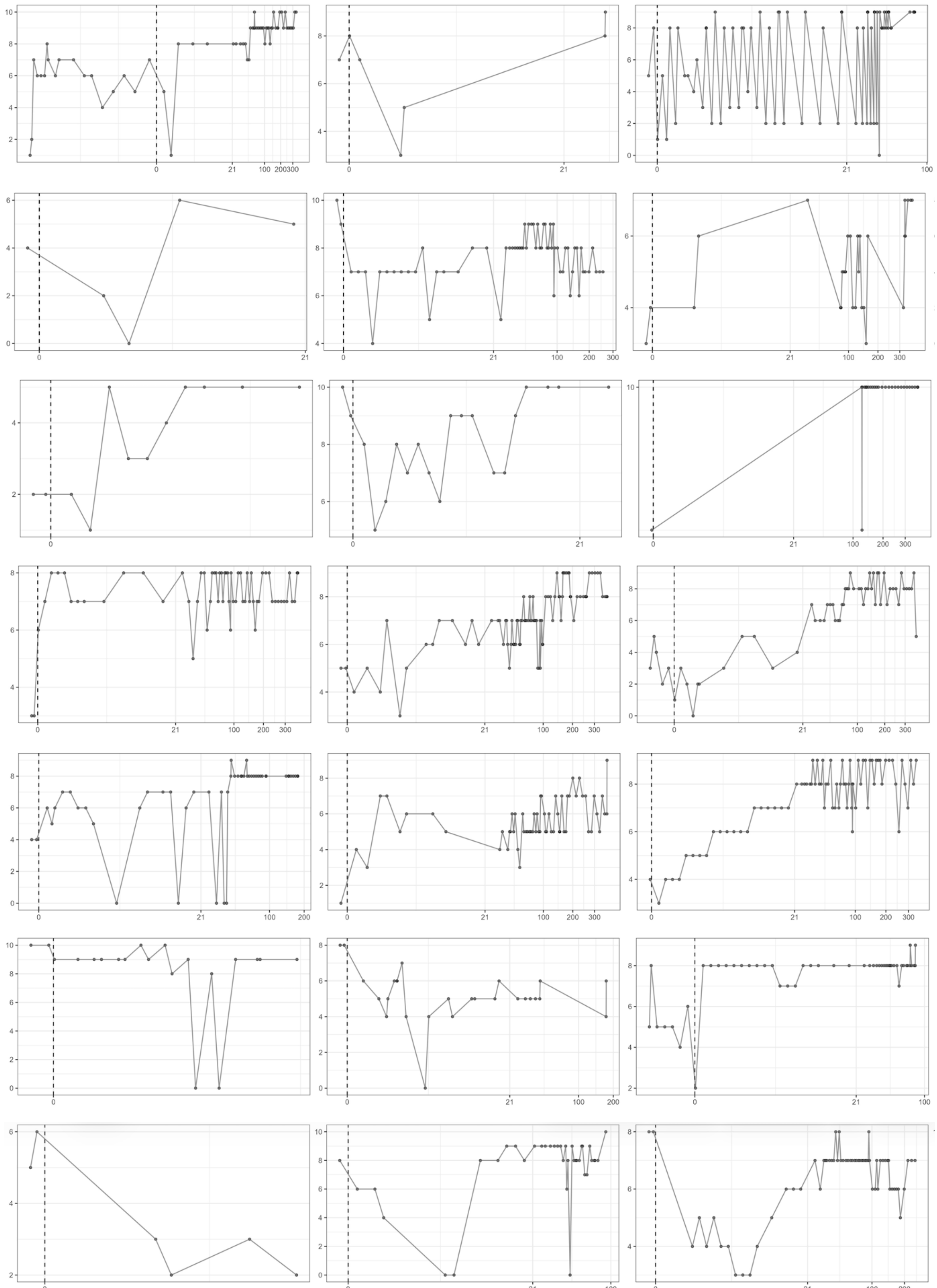

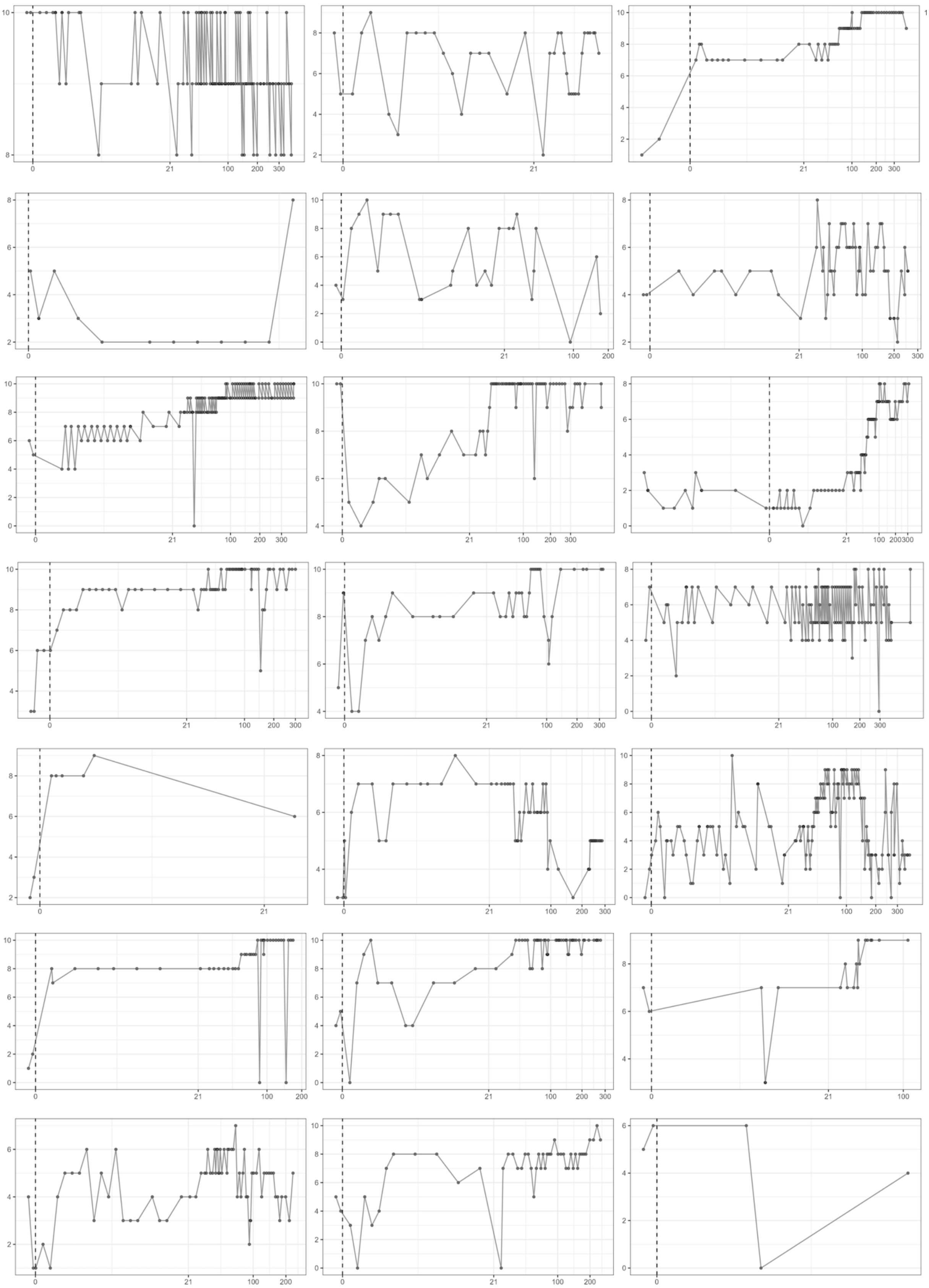

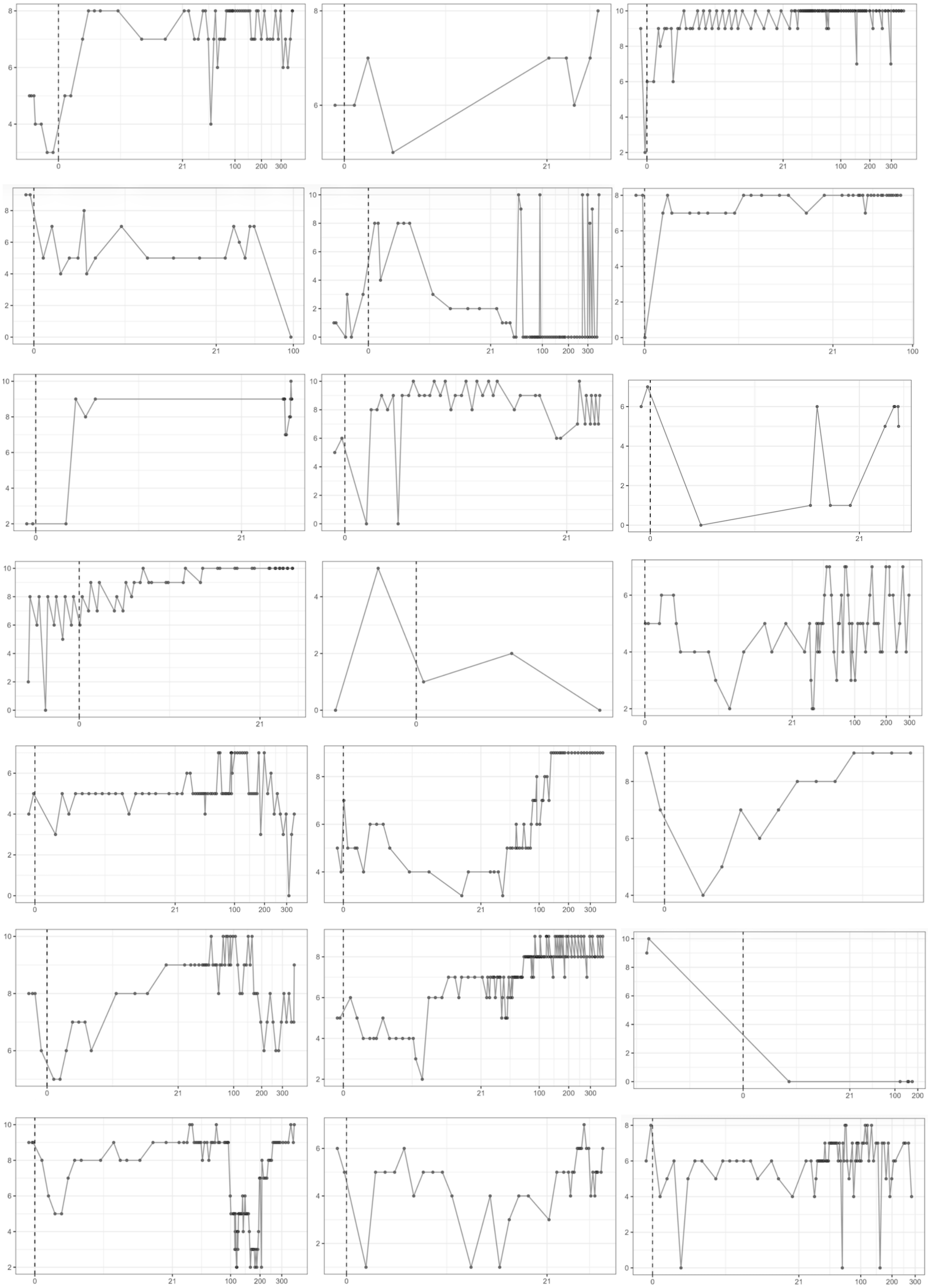

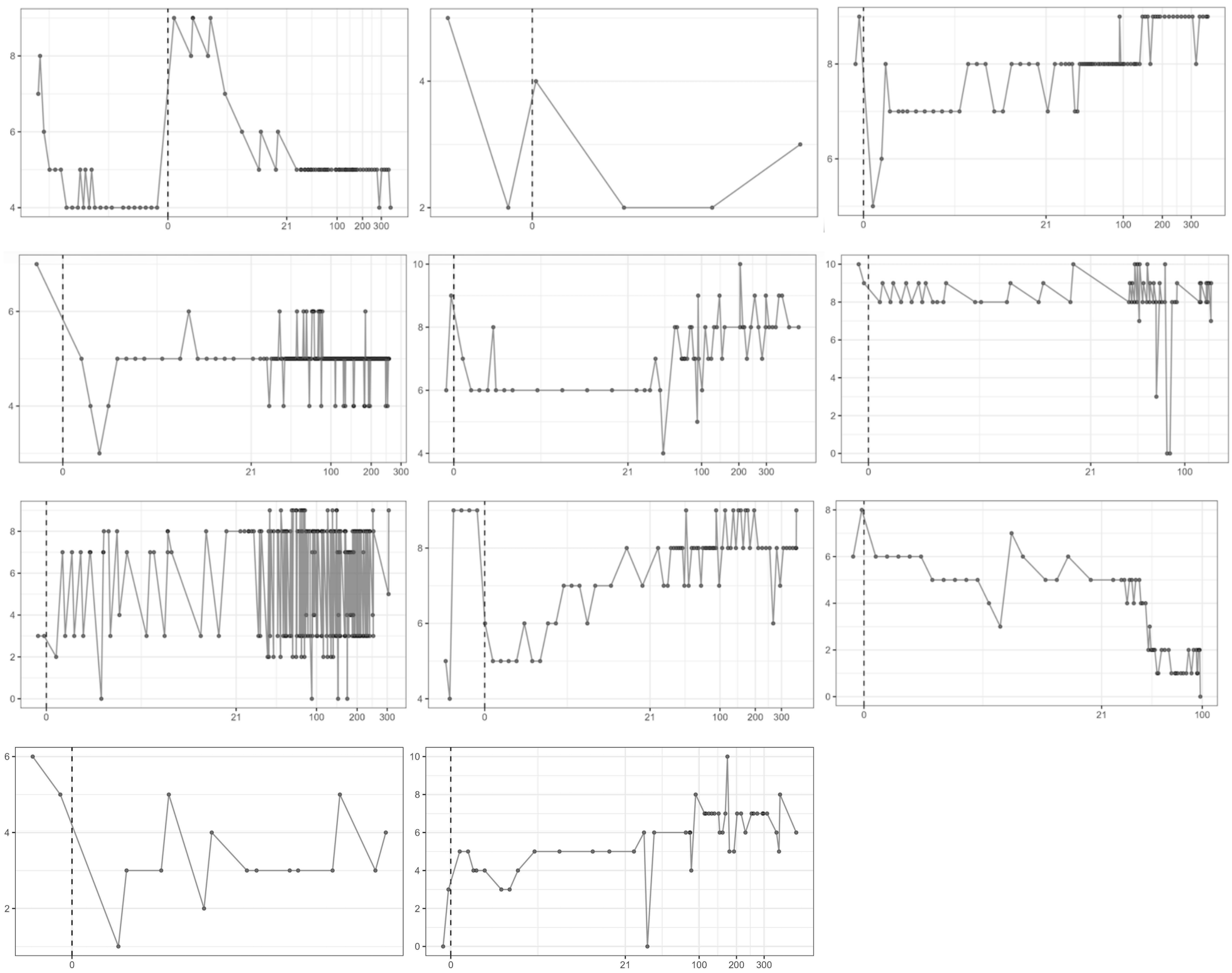

Supplement: Supplementary file 1 [file jmir-v28-e100934-s001.pdf]
