# Supplementary material for: Smartphone-Based Monitoring of Quality of Life and Adverse Events After Neurosurgery: Prospective Cohort Study
Source: J Med Internet Res. 2026 Sep 9;28:e100934. doi: 10.2196/100934 (PMC13557324; doi:10.2196/100934)
Supplement: Supplementary file 2 [file jmir-v28-e100934-s002.pdf]

Checklist 1 – mERA Checklist

Criteria	Item No.	Description	Reported Section in Manuscript
Rationale/scientific background	1	Mentions previous studies or scientific documentation in a similar or different context, which have attempted to answer the question. States the rationale for the study.	**INTRODUCTION**
Objectives/hypotheses	2	Specific objectives of the study are stated. If a hypothesis is being tested- it is clearly stated.	**METHODS –STUDY DESIGN**
Logic Model/Theoretical framework	3	Theoretical framework/logic model depicts how the intervention influences the primary study outcome, and the pathway to the outcome. If the intervention is targeting behavior change, appropriate behavior change theory is described.	**INTRODUCTION**
Study design	4	A description of the study design and how it was arrived at is presented.	**METHODS – STUDY DESIGN**
Outcomes	5	Clearly defined primary outcome measures to meet study objectives. Secondary outcome measures should be mentioned, if relevant.	**METHODS – OUTCOMES, SAMPLING AND BIAS**
Data collection methods	6	Description of data collection methods is provided. This could include description of the study tools/survey questionnaires/interview guides.	**METHODS – ELIGIBILITY CRITERIA AND STUDY PROCEDURE**
Participant eligibility	7	Eligibility criteria for participants is described.	**METHODS – ELIGIBILITY CRITERIA AND STUDY PROCEDURE**
Participant recruitment	8	Description of how the study participants were recruited into the study. Might include self-selection, health facility based recruitment, community-based recruitment, among others.	**METHODS – DATA COLLECTION AND MANAGEMENT**
Bias	9	The risk of biases is reported. Key examples of bias include recall bias (error in the accuracy or completeness of recollections), selection bias (error individuals or groups to take part), etc.	**METHODS – OUTCOMES, SAMPLING AND BIAS**

Sampling	10	How was the sample size determined? Was attrition accounted for, if relevant to study design?	**METHODS – OUTCOMES, SAMPLING AND BIAS**
Setting and locations	11	Description of the study population, as well as details on geographic area and context. Data could be collected in a sub-sample of a geographic area/ population where the intervention is being implemented. This concept refers to details on the population/context from which data were collected.	**METHODS – STUDY DESIGN**
Comparator	12	Describes use of a comparison group. For studies that do not use a comparator, this would not be relevant. Please note that before/ after comparisons are also valid and should be noted.	**METHODS – OUTCOMES, SAMPLING AND BIAS**
Data sources/measurement	13	Describes the source of data for each variable of interest. Data source may include individuals, databases, search engines etc.	**METHODS – ELIGIBILITY CRITERIA AND STUDY PROCEDURE**
Enrollment	14	Enrollment procedures are described. The numbers of participants screened for eligibility, found to be eligible or not eligible, declined to be enrolled, and enrolled in the study are mentioned, as relevant	**RESULTS – PATIENT INCLUSION AND BASELINE CHARACTERISTICS**
Description of study population	15	Demographic and/or clinical characteristics of participants in each study cohort are described	**RESULTS – PATIENT INCLUSION AND BASELINE CHARACTERISTICS**
Reporting on outcomes	16	For each primary and secondary outcome, study findings are presented	**RESULTS**
Summary of evidence	17	General interpretation of the results in the context of current evidence and current theory is provided.	**DISCUSSION – KEY RESULTS**
Limitations	18	Discussion of study limitations. This should address sources of potential biases and imprecision.	**DISCUSSION – LIMITATIONS**
Generalizability	19	The applicability of study findings to other settings is discussed. Examples might include a discussion on study population, characteristics of the intervention, incentives, and compliance rates in other contexts.	**DISCUSSION – LIMITATIONS**

Conclusions/interpretation	20	Interpretation of the results. Discussion of barriers and/or opportunities relating to policy, programs or research is included	**CONCLUSION and DISCUSSION – INTERPRETATION**
Funding	21	Sources of funding and role of funders is mentioned	**ACKNOWLEDGMENTS**
Ethical considerations	22	The process of reviewing the ethical issues related to participant enrollment, obtaining consent and preservation of confidentiality are addressed.	**METHODS – ETHICAL CONSIDERATIONS**
Competing interests	23	Clear description of conflict of interest, if any is mentioned	**NO COMPETING INTERESTS**
Confounding	24	The risk of confounding and any methods used to address it are reported	**METHODS – STATISTICAL ANALYSIS**
Statistical methods	25	Methods for primary and additional analyses, such as subgroup analyses and adjusted analyses are described	**METHODS – STATISTICAL ANALYSIS**
Mising data	26	Methods for dealing with missing data due to incomplete surveys are reported. This refers to how the data for missing, non-response and other variables were handled.	**METHODS – STATISTICAL ANALYSIS**
Analytical methods	27	Analytical methods are clearly described. In-depth description of the analysis process and how categories/themes were derived.	**METHODS – STATISTICAL ANALYSIS (quantitative equivalent)**
Data Validation	28	Discusses use of triangulation, member checking (respondent validation), search for negative cases, or other procedures. Reporting on cross verification from 2 or more sources, inter-rater reliability should be included, if relevant.	**METHODS – DATA COLLECTION AND MANAGEMENT**
Reflexivity of account provided	29	Researchers role and relationship to the respondent, wording and phrasing of questions and other factors that might have elicited a biased response are described.	**NOT APPLICABLE**
