# Supplementary material for: Smartphone-Based Monitoring of Quality of Life and Adverse Events After Neurosurgery: Prospective Cohort Study
Source: J Med Internet Res. 2026 Sep 9;28:e100934. doi: 10.2196/100934 (PMC13557324; doi:10.2196/100934)
Supplement: Supplementary file 3 [file jmir-v28-e100934-s003.pdf]

Checklist 2 – STROBE Checklist

	Item No	Recommendation
**Title and abstract**	1	(*a*) Indicate the study's design with a commonly used term in the title or the abstract **ABSTRACT**
		(*b*) Provide in the abstract an informative and balanced summary of what was done and what was found **ABSTRACT**
**Introduction**		
Background/rationale	2	Explain the scientific background and rationale for the investigation being reported **INTRODUCTION, PARAGRAPHS 1-3**
Objectives	3	State specific objectives, including any prespecified hypotheses **INTRODUCTION, PARAGRAPH 5**
**Methods**		
Study design	4	Present key elements of study design early in the paper **METHODS – STUDY DESIGN**
Setting	5	Describe the setting, locations, and relevant dates, including periods of recruitment, exposure, follow-up, and data collection **METHODS – STUDY DESIGN and ELIGIBILITY CRITERIA AND STUDY PROCEDURE and DATA COLLECTION AND MANAGEMENT**
Participants	6	(*a*) *Cohort study*—Give the eligibility criteria, and the sources and methods of selection of participants. Describe methods of follow-up<br><br>*Case-control study*—Give the eligibility criteria, and the sources and methods of case ascertainment and control selection. Give the rationale for the choice of cases and controls<br><br>*Cross-sectional study*—Give the eligibility criteria, and the sources and methods of selection of participants<br><br>**METHODS – ELIGIBILITY CRITERIA AND STUDY PROCEDURE and DATA COLLECTION AND MANAGEMENT**
Variables	7	Clearly define all outcomes, exposures, predictors, potential confounders, and effect modifiers. Give diagnostic criteria, if applicable **METHODS – OUTCOMES, SAMPLING AND BIAS and STATISTICAL ANALYSIS**
Data sources/ measurement	8*	For each variable of interest, give sources of data and details of methods of assessment (measurement). Describe comparability of assessment methods if there is more than one group **METHODS – ELIGIBILITY CRITERIA AND STUDY PROCEDURE and DATA COLLECTION AND MANAGEMENT**
Bias	9	Describe any efforts to address potential sources of bias **METHODS – OUTCOMES, SAMPLING AND BIAS**
Study size	10	Explain how the study size was arrived at **METHODS – OUTCOMES, SAMPLING AND BIAS**

Quantitative variables	11	Explain how quantitative variables were handled in the analyses. If applicable, describe which groupings were chosen and why **METHODS – STATISTICAL ANALYSIS**
Statistical methods	12	(*a*) Describe all statistical methods, including those used to control for confounding **METHODS – STATISTICAL ANALYSIS**
		(*b*) Describe any methods used to examine subgroups and interactions **METHODS – STATISTICAL ANALYSIS**
		(*c*) Explain how missing data were addressed **METHODS – STATISTICAL ANALYSIS**
		(*d*) *Cohort study*—If applicable, explain how loss to follow-up was addressed<br>*Case-control study*—If applicable, explain how matching of cases and controls was addressed<br>*Cross-sectional study*—If applicable, describe analytical methods taking account of sampling strategy<br>**METHODS – STATISTICAL ANALYSIS**
		(*e*) Describe any sensitivity analyses **NOT APPLICABLE**
**Results**		
Participants	13*	(a) Report numbers of individuals at each stage of study—eg numbers potentially eligible, examined for eligibility, confirmed eligible, included in the study, completing follow-up, and analysed **RESULTS – PATIENT INCLUSION AND BASELINE CHARACTERISTICS**
		(b) Give reasons for non-participation at each stage **RESULTS – PATIENT INCLUSION AND BASELINE CHARACTERISTICS**
		(c) Consider use of a flow diagram **NOT APPLICABLE**
Descriptive data	14*	(a) Give characteristics of study participants (eg demographic, clinical, social) and information on exposures and potential confounders. **RESULTS – PATIENT INCLUSION AND BASELINE CHARACTERISTICS and DATA COMPLETENESS**
		(b) Indicate number of participants with missing data for each variable of interest **RESULTS – PATIENT INCLUSION AND BASELINE CHARACTERISTICS and DATA COMPLETENESS**
		(c) *Cohort study*—Summarise follow-up time (eg, average and total amount) **RESULTS – PATIENT INCLUSION AND BASELINE CHARACTERISTICS and DATA COMPLETENESS**
Outcome data	15*	*Cohort study*—Report numbers of outcome events or summary measures over time
		Case-control study—Report numbers in each exposure category, or summary measures of exposure
		Cross-sectional study—Report numbers of outcome events or summary measures

		**RESULTS – ADVERSE EVENT REPORTING**
Main results	16	(*a*) Give unadjusted estimates and, if applicable, confounder-adjusted estimates and their precision (eg, 95% confidence interval). Make clear which confounders were adjusted for and why they were included
		(*b*) Report category boundaries when continuous variables were categorized
		(*c*) If relevant, consider translating estimates of relative risk into absolute risk for a meaningful time period<br>**RESULTS – MIXED-EFFECTS MODELS**
Other analyses	17	Report other analyses done—eg analyses of subgroups and interactions, and sensitivity analyses **RESULTS – ADVERSE EVENT REPORTING // NO SENSITIVITY ANALYSIS PERFORMED**
**Discussion**		
Key results	18	Summarise key results with reference to study objectives **DISCUSSION – KEY RESULTS**
Limitations	19	Discuss limitations of the study, taking into account sources of potential bias or imprecision. Discuss both direction and magnitude of any potential bias **DISCUSSION - LIMITATIONS**
Interpretation	20	Give a cautious overall interpretation of results considering objectives, limitations, multiplicity of analyses, results from similar studies, and other relevant evidence **DISCUSSION – INTERPRETATION**
Generalisability	21	Discuss the generalisability (external validity) of the study results **DISCUSSION – LIMITATIONS**
**Other information**		
Funding	22	Give the source of funding and the role of the funders for the present study and, if applicable, for the original study on which the present article is based **ACKNOWLEDGMENTS**
